# Determinants of false-positive plasma free metanephrines and methoxytyramine: the role of medications and chronic disease

**DOI:** 10.1530/ERC-25-0549

**Published:** 2026-05-12

**Authors:** Lara M Knigge, Kristin Potthoff, Georgiana Constantinescu, Sybille Fuld, Karolina Zawadzka, Ermal Tahirukaj, Stefan Bornstein, Jacques W M Lenders, Graeme Eisenhofer, Christina Pamporaki

**Affiliations:** ^1^ Department of Medicine ΙΙI, University Hospital Carl Gustav Carus at The TU Dresden, Dresden, Germany; ^2^ Grigore T Popa University of Medicine and Pharmacy, Iaşi, Romania; ^3^ Department of Endocrinology, Oncological Endocrinology, Nuclear Medicine and Internal Medicine, University Hospital in Krakow, Krakow, Poland; ^4^ Doctoral School of Medical and Health Sciences, Jagiellonian University Medical College, Krakow, Poland; ^5^ Department of Internal Medicine, Radboud University Medical Center, Nijmegen, The Netherlands

**Keywords:** phaeochromocytoma, paraganglioma, normetanephrine, metanephrine, medication, false-positive results, autonomic nervous system

## Abstract

Measurements of plasma free metanephrines provide a highly sensitive test for the diagnosis of pheochromocytoma/paraganglioma (PPGL). However, false-positive results remain common, posing a diagnostic dilemma for clinicians. The aim of this study was to determine whether chronic illness and commonly prescribed drugs can falsely elevate plasma free metanephrines. This retrospective study included 460 patients without PPGL. Disease exclusion was based on negative imaging or histopathological findings, or negative biochemistry at follow-up. Sex, age, plasma free normetanephrine, metanephrine, and methoxytyramine, the presence or absence of cardiometabolic comorbidities and/or chronic illness (CCI), and all prescribed medications at study inclusion were reported. In four patients under treatment with norepinephrine reuptake blockers (NRBs), metanephrines were measured at baseline and after short-term discontinuation of the drug. Among the 460 patients without PPGL, 88% (407/460) were receiving at least one medication. In this cohort, false-positive elevations of plasma free metanephrines were rare (7%). Multivariable analysis identified NRBs as the only drugs independently associated with increased plasma free normetanephrine levels (*P* < 0.001), in a dose-dependent manner. Importantly, plasma free methoxytyramine concentrations were independently influenced by NRBs, antipsychotics, and nicotine use (*P* < 0.001). Paired analyses in a subset of four patients before and after short-term NRB withdrawal showed normalization of plasma free normetanephrine concentrations. Plasma free metanephrines show high diagnostic reliability with minimal interference from CCI or commonly used medications. However, NRBs can increase normetanephrine and methoxytyramine concentrations, particularly at higher doses, and thus, their use requires cautious interpretation of plasma free metanephrine measurements.

## Introduction

Pheochromocytomas and paragangliomas (PPGLs) are rare neuroendocrine neoplasms originating from chromaffin cells of the adrenal medulla or extra-adrenal paraganglia (1). The clinical presentation of PPGL is often nonspecific, necessitating reliance on biochemical testing for diagnosis. The Current Endocrine Society guidelines recommend liquid chromatography measurements of plasma free or 24-h urinary fractionated normetanephrine and metanephrine as the screening tests of choice (2, 3). Evidence from the prospective PMT study demonstrated that plasma free metanephrines exhibit superior diagnostic performance compared with 24-h urinary metanephrines for patients at high risk of PPGL (4). That study also demonstrated that inclusion of 3-methoxytyramine in the plasma panel is particularly valuable for identifying dopamine-producing tumors (5).

Despite the excellent diagnostic performance of plasma free metanephrines and methoxytyramine, due to the low prevalence of disease among patients tested for PPGL (<1%), it can be expected that false-positive results will predominate at least six-fold more frequently than true-positive results. If preanalytical precautions are followed (6, 7, 8), false-positive results are mainly associated with mild elevations of plasma normetanephrine due to some form of sympathoadrenal activation (9).

There are numerous disorders associated with sympathoadrenal activation, increases in catecholamines and potential for false-positive test results of catecholamine metabolites depending on the nature and severity of chronic illness (10). Pharmacodynamic factors such as drugs with direct actions on the autonomic nervous system are also important to consider as they account for approximately 20% of all false-positive findings (11). Indeed, it was previously shown that drugs that block norepinephrine reuptake (NRBs), such as tricyclic antidepressants (TCAs) and serotonin–norepinephrine reuptake inhibitors (SNRIs), increase significantly the frequency of false-positive results for urinary (12) and plasma metanephrines in individuals without PPGL (13). Similarly, atypical antipsychotics, including quetiapine, clozapine, and risperidone, as well as nicotine use (14, 15, 16) have been associated with false-positive elevations of plasma free normetanephrine. In contrast, selective serotonin reuptake inhibitors (SSRIs) do not appear to elevate plasma free metanephrines (12, 13). With regard to antihypertensive drugs, apart from phenoxybenzamine, there is little evidence to suggest that antihypertensive medications are a major source of false-positive results in urinary or plasma free metabolites (12, 17).

However, all aforementioned studies have important limitations. Many studies examined small cohorts (15, 16) without comprehensively assessing all agents with potential interference (14, 17), while others failed to adequately control for chronic illness (13, 16) or adhere to key preanalytical factors such as fasting and supine position prior to blood sampling (17). Some studies relied only on catecholamines (15) and others relied only on urinary (18) or fractionated (12) rather than plasma free metanephrines and most of them were affected by selection bias due to inconsistent inclusion criteria. Crucially, none of the aforementioned studies used multivariable analysis, which is essential for disentangling the independent effects of medications and chronic illness on plasma free metanephrine levels. Thus, the objective of the present study was to assess whether chronic illness and commonly prescribed medications with potential effects on the sympathetic nervous system or catecholamine metabolism may lead to false-positive test results for plasma free normetanephrine, metanephrine, and methoxytyramine.

## Methods

### Patients

This study included retrospective data from 460 patients tested for PPGL, enrolled under the PMT clinical protocol at University Hospital, Dresden, Germany. All patients provided written informed consent, and the protocol was approved by the local ethics committee (Ethikkommission an der Technischen Universität Dresden). Patients were enrolled in the PMT study if they fulfilled at least one of the following criteria: i) signs and/or symptoms of catecholamine excess, ii) pathogenic variants in PPGL susceptibility genes, iii) incidental adrenal mass, or iv) previous history of PPGL. Exclusion of PPGL was established according to the following criteria: i) negative biochemical test results and/or imaging studies at least one year after inclusion in the study, ii) an alternative histopathological diagnosis for a resected tumor, or iii) negative imaging studies and/or negative clonidine suppression test in patients with positive screening test results.

Collected clinical data included date of inclusion, age, sex, reason for testing, the presence or absence of comorbidities and/or chronic illness (CCI), nicotine use, and a listing of all medications taken. Doses of medication were reported and categorized as low, intermediate, or high according to the FDA drug instructions (https://nctr-crs.fda.gov/fdalabel/ui/search). Data also included measurements of plasma free normetanephrine, metanephrine, and methoxytyramine. For four patients under treatment with NRBs, metabolites were measured at baseline and after discontinuation of the drug for at least five days, taking into consideration the half-life of each individual medication (Supplementary Table 1 (see section on Supplementary materials given at the end of the article)).

### Comorbidity score

To evaluate whether CCI may lead to false-positive results of plasma free metabolites, we developed a comorbidity score based on five major disease categories with potential effect on the sympathetic nervous system: metabolic syndrome, cardiovascular disease, chronic kidney disease, neurological disease, and chronic pulmonary disease. The metabolic syndrome group included patients with type 2 diabetes mellitus, obesity, hepatic steatosis, and/or hyperlipoproteinemia. The cardiovascular disease category comprised hypertensive heart disease, chronic heart failure, coronary artery disease, prior myocardial infarction, peripheral artery disease, and/or pulmonary embolism. Patients with stage II–V chronic kidney disease were classified under the chronic kidney disease group. The neurological group included patients with a history of transient ischemic attack, cerebellar infarction, chronic migraine, and/or epilepsy. Finally, patients diagnosed with asthma or chronic obstructive pulmonary disease were categorized within the chronic pulmonary disease group. The diagnostic criteria for each comorbidity are summarized in Supplementary Table 2. For each category, one point was assigned, yielding a maximum comorbidity score of five points for each patient.

### Biochemical analysis

Blood samples were collected after overnight fasting and at least 20 min of supine rest in heparinized tubes and kept on ice until centrifugation to separate plasma. Plasma free normetanephrine, metanephrine, and methoxytyramine were measured by liquid chromatography with tandem mass spectrometry (LC-MS/MS) according to a previously described method (19). Upper cutoffs for plasma normetanephrine were age-specific as described elsewhere (20). Upper cutoffs for metanephrine and methoxytyramine were 84 pg/mL and 17 pg/mL, respectively.

### Statistical analysis

Continuous variables are shown as median and interquartile range. Categorical parameters were analyzed using the chi-square test. A comparison of continuous parameters was performed with the Mann–Whitney U test. Since most patients on medications received a combination of two or more potentially interfering agents, multivariable analysis was used for all patients to evaluate the impacts of those medications. Pairwise analysis was used for four patients who were taking a single interfering medication at one sampling time point and no interfering medications at another. Statistical analysis was performed using JMP pro statistical software package, version 15. *P* < 0.05 was considered statistically significant.

## Results

### Patient characteristics

Among 460 patients without PPGL included in the study, 407 (88%) were receiving one or more medications (Table 1). Metabolic syndrome-related conditions were the most prevalent comorbidities, including hyperlipoproteinemia (20.0%), obesity (18.7%), and type 2 diabetes mellitus (18.6%). Cardiovascular diseases were less frequent and mainly included hypertensive heart disease (7.2%) and chronic heart failure (3.3%). Finally, chronic pulmonary diseases (e.g. sleep apnea 4.6%), chronic kidney disease, and neurological comorbidities occurred less commonly (Supplementary Table 3).

**Table 1:** Characteristics and habits of patients with and without medications. *P*<0.05 values are presented in bold.

	No medication	Medication	*P* value
Number	12% (53/460)	88% (407/460)	
Age	46 (18–78)	55 (19–84)	**0.003**
Sex (female)	42% (22/53)	49% (200/407)	0.162
Nicotine consumption*	24% (8/34)	13% (52/402)	0.218
Reason for testing			0.342
Signs and symptoms	53% (28/53)	64% (262/407)	
Incidentalomas	28% (15/53)	28% (115/407)
Hereditary predisposition/previous history of PPGL	19% (10/53)	7% (30/407)	
Comorbidities/chronic illness			
Metabolic syndrome^†^	25% (13/53)	45% (183/407)	**0.002**
Cardiovascular disease^‡^	0% (0/53)	14% (58/407)	**0.001**
CKD^§^	0% (0/53)	5% (22/407)	0.058
Neurological disease^║^	6% (3/53)	12% (50/407)	0.127
COPD/asthma	4% (2/53)	7% (29/407)	0.307
Comorbidity score	0.3	0.8	**0.001**
Biochemistry			
Plasma free normetanephrine (pg/mL)	80 (35–219)	88 (11–365)	0.142
Plasma free metanephrine (pg/mL)	27 (12–244)	28.5 (1–145)	0.766
Plasma free methoxytyramine (pg/mL)	5.6 (2.2–14)	5.8 (1.5–43)	0.219
Rates of false-positive results			
Normetanephrine false positives	2% (1/53)	7% (27/407)	0.142
Metanephrine false positives	0% (0/53)	1% (5/407)	0.600
Methoxytyramine false positives	0% (0/53)	1% (5/407)	0.198

* For 127 patients, 19 without and 108 with medication, data regarding nicotine consumption were not available.

^†^ Includes diabetes mellitus type II, obesity, hepatic steatosis, and hyperlipoproteinemia.

^‡^ Includes chronic heart disease, hypertensive heart disease, myocardial infarction, pulmonary artery infarction, and peripheral artery disease.

^§^ Includes chronic kidney disease.

^║^ Includes transient ischemic attack, cerebellar infarction, stroke, and migraines. Data are presented as % or median (IQR).

As anticipated, patients on medications were older (*P* = 0.003) and exhibited a higher comorbidity score (*P* < 0.001) compared to those without medical treatment. In particular, patients on medications presented with a higher prevalence of metabolic (*P* = 0.002) and cardiovascular disease (*P* = 0.001) compared to those without medical treatment. Plasma concentrations of free normetanephrine, metanephrine, and methoxytyramine did not differ significantly between the two groups. Rates of false-positive test results in the entire cohort were 7% (33/460). In particular, rates of false-positive results for plasma free normetanephrine, metanephrine, and methoxytyramine were 6% (28/460), 1% (5/460), and 1% (5/460), respectively (Table 1).

Among all patients, 22 (5%) were under treatment with NRBs, including SNRIs and TCAs (Table 2). Fourteen patients (3%) were under treatment with SSRIs, whereas 24 (5%) received antipsychotics. Among 366 patients under antihypertensive treatment, 177 patients were under treatment with diuretics, 184 with beta-blockers, 197 with calcium channel blockers, 129 with angiotensin-converting enzyme inhibitors, 170 with angiotensin receptor blockers, 65 with alpha blockers, 61 with central alpha agonists, 15 with direct vasodilators, and five with renin inhibitors. Importantly, 278 patients were treated with more than one antihypertensive drug.

**Table 2:** Plasma concentrations of plasma free normetanephrine, metanephrine, and methoxytyramine in the absence or presence of chronic illness/comorbidities, nicotine, and different classes of specific medication. *P*<0.05 values are presented in bold.

	Absence	Presence	*P* value	*P* value MV*
CCI, *n*	200	260		
Normetanephrine	81 (11–229)	95 (19–365)	**0.004**	
Metanephrine	30 (5–145)	28 (1–103)	**0.035**	
Methoxytyramine	5.6 (2–12)	5.9 (2–43)	**0.001**	
Nicotine, *n*	273^†^	60		
Normetanephrine	87 (11–241)	92 (19–202)	0.350	-
Metanephrine	27 (1–94)	35 (10–103)	**0.008**	
Methoxytyramine	5.6 (2–21)	6.4 (2–43)	**0.001**	**0.001**
NRBs, *n*	438	22		
Normetanephrine	87 (11–365)	137 (51–219)	**0.001**	**0.001**
Metanephrine	29 (1–145)	19 (10–81)	0.571	-
Methoxytyramine	5.7 (2–43)	10 (4–26)	**0.001**	**0.001**
SSRIs, *n*	446	14		
Normetanephrine	87 (22–365)	102 (22–201)	0.147	-
Metanephrine	29 (1–145)	21 (5–64)	0.342	-
Methoxytyramine	5.8 (2–26)	4.7 (4–43)	**0.004**	
Antipsychotics, *n*	436	24		
Normetanephrine	87 (11–365)	98 (28–219)	0.851	-
Metanephrine	28 (1–145)	23 (17–75)	0.352	-
Methoxytyramine	5.7 (2–43)	7 (3–26)	**0.001**	**0.001**
Diuretics, *n*	283	177		
Normetanephrine	84 (11–365)	95 (23–283)	0.094	-
Metanephrine	29 (1–94)	27 (2–145)	0.098	-
Methoxytyramine	5.6 (2–25)	6.2 (2–43)	0.053	-
β-adrenoreceptor blockers, *n*	276	184		
Normetanephrine	85 (11–219)	91 (19–365)	0.075	
Metanephrine	28 (1–103)	28 (2–145)	0.727	-
Methoxytyramine	5.5 (1.5–24)	6.3 (2–43)	0.067	
Calcium channel blockers, *n*	263	197		
Normetanephrine	80 (22–243)	95 (11–365)	**0.001**	
Metanephrine	29 (1–94)	27 (2–145)	0.381	-
Methoxytyramine	5.3 (1.5–20.6)	6.2 (2–43)	**0.024**	
Angiotensin-converting enzyme inhibitors, *n*	331	129		
Normetanephrine	85 (19–283)	93 (11–365)	**0.015**	
Metanephrine	27 (2–145)	30 (1–94)	0.802	-
Methoxytyramine	5.7 (1.5–24)	6 (2–43)	**0.007**	
Angiotensin receptor blockers, *n*	290	170		
Normetanephrine	85 (11–365)	91.5 (30–283)	0.494	-
Metanephrine	29 (1–103)	27 (2–145)	0.781	**-**
Methoxytyramine	5.8 (1.5–43)	5.7 (2–17)	0.736	-
Alpha blockers, *n*	395	65		
Normetanephrine	86 (11–365)	95 (30–292)	**0.041**	
Metanephrine	29 (1–145)	26 (5–103)	**0.023**	
Methoxytyramine	5.7 (1.5–43)	6.8 (2–25)	0.298	-
Central alpha agonists, *n*	399	61		
Normetanephrine	87 (11–365)	89 (30–292)	0.438	-
Metanephrine	32 (1–145)	30 (3–94)	0.065	-
Methoxytyramine	5.7 (1.5–23)	6.8 (2–43)	0.110	-
Direct vasodilators, *n*	445	15		
Normetanephrine	87 (11–365)	88 (35–151)	0.482	-
Metanephrine	28.5 (1–145)	26.7 (11–69)	0.564	-
Methoxytyramine	5.7 (1.5–43)	7.4 (4–10.6)	0.407	**-**

* MV, multivariable analysis.

^†^ For 127 patients, information on smoking was not available; CCI, comorbidities/chronic illness; NRBs, norepinephrine reuptake blockers; SSRIs, selective serotonin reuptake inhibitors.

### Rates of false-positive results

Among the 28 patients with false-positive plasma free normetanephrine concentrations, 24 (86%) had CCI and eight (29%) were smokers. All smokers were under one or more medications. Five patients were treated with NRBs, two with SSRIs, and two with antipsychotics. The vast majority were receiving one or more antihypertensive medications (Table 3). Among the five patients with false-positive concentrations of plasma free metanephrine, three (60%) had CCI and one was a smoker. None were treated with neuropsychiatric medications, but all were on one or more antihypertensive agents. Finally, all five patients with false-positive results for methoxytyramine had CCI, and three (60%) were smokers. Three (60%) were treated with NRBs, one (20%) with an SSRI, and two (40%) with antipsychotics. All patients were on one or more antihypertensive medications (Table 3).

**Table 3:** Clinical conditions and medications observed in patients with false-positive results for plasma free normetanephrine, metanephrine, and methoxytyramine.

	False-positive results		
	Normetanephrine,	Metanephrine,	Methoxytyramine,
	*n* = 28	*n* = 5	*n* = 5
CCI	24 (86%)	3 (60%)	5 (100%)
Nicotine	8 (29%)	1 (20%)	3 (60%)
NRBs	5 (18%)	-	3 (60%)
SSRIs	2 (7%)	-	1 (20%)
Antipsychotics	2 (7%)	-	2 (40%)
Diuretics	17 (60%)	4 (80%)	4 (80%)
β-blockers	14 (50%)	3 (60%)	3 (60%)
Calcium channel blockers	17 (60%)	4 (80%)	3 (60%)
Angiotensin-converting enzyme inhibitors	14 (50%)	2 (40%)	3 (60%)
Angiotensin receptor blockers	32% (9/28)	40% (2/5)	20% (1/5)
Alpha blockers	18% (5/28)	40% (2/5)	20% (1/5)
Central agonists	14% (4/28)	60% (3/5)	40% (2/5)
Vasodilators	-	-	-

CCI, comorbidities/chronic illness; NRBs, norepinephrine reuptake blockers; SSRIs, selective serotonin reuptake inhibitors.

### Impact of medication on plasma free metabolites: multivariable analysis

Among all patients without PPGL, multivariable regression analysis with adjustment for age, sex, and comorbidity score demonstrated that only NRBs were significantly associated with increased plasma free normetanephrine concentrations (*P* < 0.001). In addition, nicotine consumption, NRBs, and antipsychotics each showed significant independent associations with plasma free methoxytyramine concentrations (*P* < 0.001). No medication class had a significant effect on plasma free metanephrine concentrations (Table 2).

### Dose-dependent impact of NRBs on plasma free metabolites

Among the 22 patients receiving NRBs, five presented with false-positive results (Tables 4 and 5). Four (80%) patients were males, mean age was 56 years, and all presented with at least one CCI. Three patients showed elevations in both plasma free normetanephrine and methoxytyramine, while two patients exhibited isolated increases in plasma free normetanephrine. Notably, two patients (patients 1 and 2) were treated with a moderate dose of TCAs and SNRIs, respectively, whereas three patients were treated with high-dose NRBs in a single (patient 3) or combination (patients 4, 5) of two NRB agents with at least one agent at a moderate or high dose (Table 5, Supplementary Table 4). None of the patients receiving low-dose NRBs demonstrated false-positive results. Furthermore, none of the 22 patients on NRBs presented false-positive results of plasma free metanephrine.

**Table 4:** Rates of false-positive results in patients under treatment with low, moderate, and high doses of norepinephrine reuptake blockers.

Patients on NRBs	Low dose	Moderate dose	High dose
Number	7	6	9
Plasma free normetanephrine			
True negatives	100% (7/7)	67% (4/6)	67% (6/9)
False positives	-	33% (2/6)	33% (3/9)
Plasma free metanephrine			
True negatives	100% (7/7)	100% (6/6)	100% (9/9)
False positives	-	-	-
Plasma free methoxytyramine			
True negatives	100% (7/7)	83% (5/6)	78% (7/9)
False positives	-	17% (1/6)	22% (2/9)

**Table 5:** Characteristics of patients under treatment with norepinephrine reuptake blockers (NRBs) and false-positive results of plasma free normetanephrine and methoxytyramine, and 24-h urinary fractionated normetanephrine and metanephrine. Values in bold are above the upper cut off.


	Patient 1	Patient 2	Patient 3	Patient 4	Patient 5
Sex	Male	Female	Male	Male	Male
Age (years)	57	52	43	68	60
Type of NRB	TCA	SNRI	SNRI	NaSSA/SNRI	SNRI and TCA
Name	Amitriptyline: 75 mg	Duloxetine: 60 mg	Duloxetine: 120 mg	Mirtazapine: 45 mg;venlafaxine: 37.5 mg	Venlafaxine: 150 mg; doxepin: 20 mg
Dose	Moderate	Moderate	High	High	High
Plasma free NMN (pg/mL)					
With NRBs	**205**	**184**	**224**	**243**	**219**
Without NRBs	161	145	117	107	-
Plasma free MN (pg/mL)					
With NRBs	39	65	19	41	**45**
Without NRBs	42	61	17	53	-
Plasma free MTY (pg/mL)					
With NRBs	**29**	6.8	**20.6**	13	**26**
Without NRBs	**30**	8	7.1	6	-
Fractionated urine NMN (iU/day)					
With NRBs	**-**	-	**4,016**	-	-
Without NRBs	**-**	-	-	**2,298**	**3,025**
Fractionated urine MN (iU/day)					
With NRBs	**-**	-	619	-	-
Without NRBs	**-**	-	-	975	735

NRB, norepinephrine reuptake blocker; TCA, tricyclic antidepressant; SNRI, serotonin–norepinephrine reuptake inhibitor; NaSSA, noradrenergic and specific serotonergic antidepressant; NMN, normetanephrine; MN, metanephrine; MTY, methoxytyramine. The upper cutoff for urinary fractionated normetanephrine was 1,950 iU/day, and that for metanephrine was 1,520 iU/day.

### Impact of NRBs on plasma free metabolites: paired analyses

**Figure 1:**
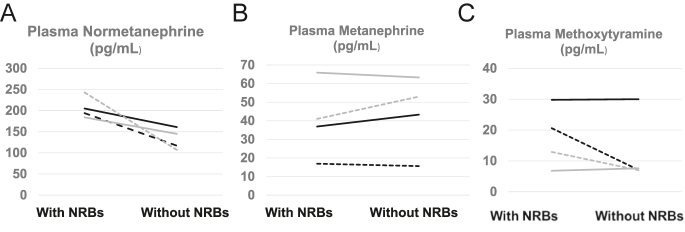
Paired analysis before and after discontinuation of NRBs of plasma concentrations of free normetanephrine (A), metanephrine (B), and methoxytyramine (C).

Paired analyses with and without NRBs were possible in four individuals (patients 1–4) who received moderate- or high-dose NRBs and initially presented with false-positive elevations of normetanephrine (Table 5). All four patients demonstrated a reduction of normetanephrine levels to below the upper cutoffs following short-term withdrawal of the medication (Table 5, Fig. 1, Supplementary Table 1). Importantly, 24-h urinary fractionated measurements of metanephrines were available for patients 3, 4, and 5. All three exhibited false-positive elevations of urinary fractionated normetanephrine, two of which (patients 4 and 5) even after washout of the suspected NRBs (Table 5). Notably, the magnitude of the increase for 24-h urinary normetanephrine was consistently greater than that observed for the corresponding plasma metabolite. Finally, it should be noted that patients 1 and 3 also exhibited initial false-positive elevations of methoxytyramine. However, normalization of methoxytyramine levels after NRB discontinuation was observed only in patient 3 (Fig. 1).

## Discussion

This is the first study based on a standardized clinical protocol to demonstrate that plasma free metanephrines constitute a diagnostic test with negligible overall interference from drug-induced activation of the sympathetic nervous system. Notably, after multivariable analysis, only NRBs were shown to significantly affect plasma free normetanephrine concentrations. This effect was most likely dose-dependent and was further confirmed by paired analyses, which demonstrated normalization of normetanephrine levels following short-term discontinuation of NRB therapy.

Catecholamines are secreted by sympathetic nerves and adrenal chromaffin cells to maintain homeostasis under physiological and pathological stress. Numerous conditions including myocardial infarction (21, 22), stroke (23, 24, 25), heart failure, hypertension, hypoxic disorders, depression, and cirrhosis (26, 27) have been associated with increased catecholamine levels and a potential for false-positive results. In contrast to previous studies, our study was unable to find an independent association between comorbidities/chronic illness (CCI) and elevations of plasma free metanephrines. It should be noted that although chronic kidney disease (CKD) was not independently associated with elevations of plasma free metabolites in this study, the vast majority of patients with CKD in our cohort had mild-to-moderate disease (stages II–III). Previous studies have shown that advanced CKD (III–V) is indeed associated with a high rate of false-positive results for plasma free normetanephrine (28), so that the use of CKD-specific reference intervals is highly recommended (28, 29).

Apart from CCI, pharmacodynamic false-positive results may arise from drugs that act directly on the autonomic nervous system. In our study, NRBs were independently associated with increased plasma free normetanephrine and methoxytyramine concentrations. NRBs have multiple mechanisms of action on the sympathetic nervous system. While they inhibit norepinephrine reuptake and increase its escape into circulation, they also markedly suppress sympathetic nerve firing (30). This suppression is mediated by central α2-adrenoceptor activation due to increased catecholamines in the brainstem (31). Consequently, short-term use of NRBs is not associated with markedly elevations of free normetanephrine in plasma, but chronic use partial adaptation may occur, shifting the balance toward increased norepinephrine escape. Therefore, chronic use of NRBs is associated with an increased likelihood of moderate elevations in plasma free normetanephrine concentrations, thereby increasing the risk of false-positive results (32). It should be appreciated, though, that variations in central α2-adrenoreceptor-mediated sympathoinhibition may explain variable responses of norepinephrine and normetanephrine to NRBs, including the exceptional cases described in the literature with prominent elevations of plasma free normetanephrine (13). The independent effect of NRBs on plasma free normetanephrine established in our study was further confirmed by paired analyses in four patients receiving NRBs who initially showed elevated plasma free normetanephrine, and normalization of false-positive results after short-term drug withdrawal.

Importantly, our study shows that the effect of NRBs on plasma free normetanephrine is likely dose-dependent. In particular, all five patients with false-positive test results were under moderate- or high-dose NRB monotherapy or combination of NRB agents, with at least one regimen in moderate or high dose. This dose-dependent effect is especially relevant for treatment with SNRIs. Indeed, it is well established that at low doses, SNRIs mainly inhibit serotonin reuptake due to higher transporter sensitivity, whereas at higher doses they also strongly block norepinephrine transporters, producing significant norepinephrine reuptake inhibition (33). Since NRBs at low doses were not associated with higher false-positive rates of normetanephrine in our study, dose reduction of NRB treatment before repeating biochemical testing, in case of positive biochemical test results, may be a practical alternative to complete medication withdrawal or imaging studies when discontinuation is not an option. Nevertheless, while preliminary observations suggest a low probability of false-positive results under low-dose NRB monotherapy, the current sample size precludes formal dose-stratified analysis. Larger, systematically designed studies will be required to address this question with appropriate statistical rigor.

Another class of psychiatric drugs previously associated with false-positive normetanephrine results is atypical antipsychotics, including quetiapine, clozapine, and risperidone (21, 34). These agents antagonize dopaminergic, adrenergic, and serotonergic receptors. The exact mechanism by which they increase norepinephrine secretion remains unclear, but α2-adrenergic and dopaminergic D2 receptor antagonism may contribute. In our study, antipsychotics were independently associated with elevations of plasma free methoxytyramine, but not of normetanephrine.

In general, antihypertensive drugs may increase plasma norepinephrine due to reflex responses to blood pressure reduction, but they have a minimal impact on plasma metanephrines (12, 17). Calcium channel blockers may variably increase false-positive plasma catecholamines without affecting metanephrines. Likewise, diuretics, ACE inhibitors, and angiotensin receptor blockers show no significant effect on plasma free metabolites. Although β-blockers can slightly increase false-positive plasma metanephrines, this effect is clinically negligible. Thus, except for phenoxybenzamine, antihypertensives rarely cause false-positive results of plasma free metanephrines (10). Our study confirms these findings, as multivariable analysis showed no independent effect of antihypertensives on plasma free normetanephrine, metanephrine, and methoxytyramine.

In our cohort, apart from NRBs and antipsychotics, methoxytyramine was also influenced by nicotine use. It should be noted, though, that all smokers with false-positive methoxytyramine results were under one or more medications and had at least one CCI. Despite the rather weak relation of methoxytyramine with smoking shown in our study, previous findings have demonstrated that nicotine strongly stimulates sympathoadrenal catecholamine release, with smoking almost doubling plasma epinephrine levels (18, 19). Thus, patients should be instructed to abstain from smoking before testing. In contrast, discontinuation of NRBs or antipsychotics is not recommended for isolated methoxytyramine elevations, as normetanephrine and metanephrine remain the clinically decisive markers for diagnosis of PPGL. Consequently, results can still be reliably interpreted in the presence of mildly elevated methoxytyramine levels in patients with potentially interfering medications.

A major strength of this study is that it is the first to assess the impact of sympathetic-related comorbidities and medications on plasma free metabolites using multivariable analysis in one of the largest cohorts of patients without PPGL. All participants were enrolled under strict inclusion criteria, and plasma free metanephrines were measured using standardized preanalytical and analytical procedures with LC-MS/MS. Importantly, PPGL was excluded in all patients using rigorous and uniform exclusion criteria. Finally, this study demonstrates the likely dose-dependent effect of NRBs and shows that short-term discontinuation of NRBs is sufficient to normalize test results.

Limitations include the small number of patients treated with NRBs and the monocentric study design. In addition, data on nicotine consumption were missing for 127 patients and medication withdrawal was feasible in only four patients. Finally, in the present manuscript, we focused exclusively on plasma free metanephrines and methoxytyramine, and therefore, 24-h urinary fractionated metanephrines were not systematically analyzed. Nevertheless, among patients receiving NRBs who showed false-positive results for plasma free metabolites, 24-h urinary fractionated measurements of metanephrines were available for three individuals. All three exhibited false elevations of urinary fractionated normetanephrine, two of which were after washout of the suspected NRBs. Notably, the magnitude of the increase was consistently greater than that observed for the corresponding plasma metabolites. Considering these findings, together with previous evidence indicating that false-positive rates are approximately 28% lower for plasma free compared with urinary fractionated normetanephrine (4), it appears unlikely that 24-h urinary fractionated metanephrine measurements provide a reliable alternative to plasma free testing in patients receiving NRB therapy for avoiding false-positive results.

## Conclusion

Plasma free metanephrines provide a diagnostic test with minimal interference from sympathetic-related chronic disease or most commonly used medications with potential effects on the sympathetic nervous system. However, NRBs can increase normetanephrine and methoxytyramine concentrations, particularly at higher doses, and therefore, a cautious interpretation of test results is required.

## Supplementary materials



## Declaration of interest

There is no conflict of interest that could be perceived as prejudicing the impartiality of the research reported.

## Funding

This work was supported by the Deutsche Forschungsgemeinschaft (314061271-TRR/CRC 205-½; to CP, GC, FA, SB, and GE), by the P.R.I.S.-Programm ‘Mehr Zeit für Wissenschaft’ of the Medical Faculty, TU Dresden (to LMK, KP, and CP), and by the Habilitationsförderung für Frauen of the Medical Faculty, TU Dresden, to CP.
