# Supplementary material for: Determinants of false-positive plasma free metanephrines and methoxytyramine: the role of medications and chronic disease
Source: Endocr Relat Cancer. 2026 May 12;33(5):e250549. doi: 10.1530/ERC-25-0549 (PMC13188186; doi:10.1530/ERC-25-0549)
Supplement: Supplementary file 1 [file supplementary_materials.pdf]

Determinants of False Positive Plasma Free Metanephrines and Methoxytyramine: The Role of Medications and Chronic Disease

Lara M. Knigge[1], Kristin Potthoff[1], Georgiana Constantinescu[1,2], Sybille Fuld[1], Karolina Zawadzka[3,4], Ermal Tahirukaj[1], Stefan Bornstein[1], Jacques W.M. Lenders[5], Graeme Eisenhofer[1], Christina Pamporaki[1]

1. Department of Medicine III, University Hospital Carl Gustav Carus at the TU Dresden, Dresden, Germany

2. Grigore T Popa University of Medicine and Pharmacy, Ias Romania

3. Department of Endocrinology, Oncological Endocrinology, Nuclera Medicine and Internal Medicine, University Hospital in Krakow, Poland

4. Doctoral School of Medical and Health Sciences, Jagiellonian University Medical College, Krakow, Poland

5. Department of Internal Medicine, Radboud University Medical Center, Nijmegen, the Netherlands

Withdrawal protocol of Norepinephrine-Reuptake Blockers (NRBs)

Medication was usually withdrawn for at least five days, taking into considering the half-life of each individual medication. In fact, approximately 5 elimination half-lives are required for >95–97% drug clearance from plasma. After 7 half-lives, >99% elimination is expected. Thus, medication wash out was based on the medication half-lives as demonstrated in the supplementary Table 1.

Supplementary Table 1. Estimated Drug Elimination and Recommended Washout Periods Based on Half-Life of NRB Antidepressants

Medication	Upper Half-Life (h)	5 Half-Lives (≈97% elimination)	7 Half-Lives (≈99% elimination)	Suggested Washout Period
**Amitriptyline**	28	140 h (5.8 days)	196 h (8.2 days)	6–8 days
**Duloxetine**	17	85 h (3.5 days)	119 h (5.0 days)	4–5 days
**Mirtazapine**	40	200 h (8.3 days)	280 h (11.7 days)	8–12 days
**Venlafaxine**	11	55 h (2.3 days)	77 h (3.2 days)	~3 days
**Doxepin**	20	100 h (4.2 days)	140 h (5.8 days)	4–6 days

Comorbidities

Comorbidities were categorized into five clinical groups. The diagnostic criteria for each comorbidity are summarized in Supplementary Table 2. Metabolic syndrome related conditions were the most prevalent, with hyperlipoproteinemia present in 20.0% of patients, type 2 diabetes mellitus in 18.6%, obesity 18.7%, and hepatic steatosis in 3.7%, while gestational diabetes was rare (0.4%). Cardiovascular diseases included hypertensive heart disease (7.2%), chronic heart failure (3.3%), thromboembolic events (0.9%), and prior myocardial infarction (0.4%). Chronic kidney disease was less frequent, with stage III renal insufficiency observed only in 3.7% of patients and other stages occurring in ≤1.1%. Neurological comorbidities comprised migraine (2.4%), transient ischemic attack (1.8%), epilepsy (1.8%), stroke (1.3%), and cerebellar infarction (0.2%). Chronic pulmonary diseases were present in a smaller proportion of the cohort, including asthma (4.6%), sleep apnoea (4.6%) and chronic obstructive pulmonary disease (2.0%).

Supplementary Table 2. Diagnostic criteria of the included Comorbidities/Chronic illnesses

Comorbidities/Chronic illness	Diagnostic Criteria
**Metabolic Syndrome**	
Diabetes mellitus type II	HbA1c, oral glucose tolerance test (oGTT), or repeated fasting glucose measurements
Obesity	BMI according to WHO classification
Steatosis hepatis	Ultrasound, CT/MRI, or mildly elevated ALT/AST
Hyperlipoproteinemia	Elevated LDL, reduced HDL, increased total cholesterol or triglycerides
**Cardiovascular disease**	
Hypertensive Heart disease	Hypertension confirmed by office blood pressure and transthoracic echocardiography
Chronic Heart failure	Transthoracic echocardiography
Coronary Heart disease	Typical angina symptoms and/or pathological:<br>• Stress ECG<br>• Stress MRI<br>• Stress echocardiography<br>• Coronary angiography<br>• Coronary CT angiography
Myocardial Infarction	Diagnosis documented in clinical or emergency/intensive care records
Peripheral arterial disease (PAD)	Ankle-brachial index (ABI) and/or duplex ultrasonography
Pulmonary Embolism	Elevated D-dimer and/or CT angiography and/or ventilation/perfusion scintigraphy
**Chronic kidney disease**	
Chronic renal insufficiency	Diagnostic clinical criteria (KDIGO-Classification)
**Neurological disease**	
Transient ischaemic attack	Acute focal neurological symptoms (<24h), MRI, CT
Stroke	Neurological symptoms (>24h), MRI, CT
Migraine	Diagnostic clinical criteria (ICHD-3)
Epilepsy	Diagnostic clinical criteria (ILAE-classification)
**Chronic pulmonary disease**	
Asthma	Diagnostic clinical criteria (GINA), Spirometry
COPD	Diagnostic clinical criteria (WHO), Spirometry
Obstructive sleep apnoea	Diagnostic clinical criteria (DGSM), Polysomnography

Supplementary Table 3. Prevalence of Comorbidities in the Study Cohort by Disease Group

Group	Comorbidity	n	%
**Metabolic Syndrome**	Diabetes mellitus II	85	18.5
	Hepatic steatosis	17	3.7
	Hyperlipoproteinemia	91	19.7
	Gestational diabetes	2	0.4
	Obesity	86	18.7
**Cardiovascular disease**	Hypertensive heart disease	33	7.2
	Chronic heart failure	15	3.3
	Heart attack	2	0.4
	Thromboembolic event	4	0.9
**Chronic kidney disease**	Chronic renal insufficiency II	5	1.1
	Chronic renal insufficiency III	17	3.7
	Chronic renal insufficiency I	1	0.2
	Chronic renal insufficiency (Stage not specified)	4	0.9
**Neurological disease**	Transient cerebral ischemia	8	1.7
	Cerebellar infarction	1	0.2
	Migraine	11	2.4
	Epilepsy	8	1.7
	Stroke	6	1.3
**Chronic pulmonary disease**	Asthma	21	4.6
	COPD	9	1.9
	Obstructive sleep apnoea	21	4.6

Dose-dependent impact of NRBs on plasma free metabolites

The dosage ranges were derived from the official U.S. Food and Drug Administration (FDA) approved prescribing information for each medication, including labeled starting doses, usual therapeutic ranges, and maximum recommended daily doses (see Table below). These values reflect regulatory-approved guidance for adult patients. In cases where patients were receiving a combination of different norepinephrine-reuptake blockers NRB regimens (e.g., TCA/SNRI or NaSSA/SNRI) with at least one regimen in moderate or high dose, the combined treatment dose was classified as “high dose”. It should be noted that, although mirtazapine (NaSSA) is pharmacologically distinct from SNRIs, both drug classes enhance noradrenergic and serotonergic neurotransmission. For this functional reason, NaSSAs were categorized within the same group. Supplementary Table 1 presents the the FDA-based dose classification of different NRBs.

Supplementary Table 4. Dose Classification of Noradrenergic Reuptake–Based Antidepressants (NRBs): FDA-Referenced Low, Moderate, and High Daily Dose Ranges

Medication	Low (starting) dose	Moderate Dose	High Dose
**TCAs**			
Amitriptyline	25-30 mg/day	75-150 mg/day	300 mg/day
Doxepin	25-30 mg/day	75-150 mg/day	300 mg/day
**SNRIs**			
Duloxetine	30 mg/day	60 mg/day	120 mg/day
Venlafaxine	75 mg/day	75-225 mg/day	225 mg/day
**NaSSA**			
Mirtazapine	15 mg/day	30 mg/day	45 mg/day

TCAs: tricyclic antidepressants, SNRIs: serotonin–norepinephrine reuptake inhibitors, NaSSA: Noradrenergic and Specific Serotonergic Antidepressant
